# Four Distinct Subgroups of Self-Injurious Behavior among Chinese Adolescents: Findings from a Latent Class Analysis

**DOI:** 10.1371/journal.pone.0158609

**Published:** 2016-07-08

**Authors:** Xiuhong Xin, Qingsen Ming, Jibiao Zhang, Yuping Wang, Mingli Liu, Shuqiao Yao

**Affiliations:** 1 Medical Psychological Institute, Second Xiangya Hospital, Central South University, Changsha, Hunan, 410011, China; 2 Department of Medical Psychology, Clinical Medical College, Ningxia Medical University, Yinchuan, Ningxia, China; 3 Mental Health Center, General Hospital of Ningxia Medical University, Yinchuan, Ningxia, China; 4 Psychology Department, School Education, Jianghan University, Wuhan, Hubei, China; 5 School of Humanities & Social Sciences, Xi’an Jiaotong University, Xi’an, China; 6 School of Education, Hunan University of Science and Technology, Xiangtan, Hunan, China; Harvard Medical School, UNITED STATES

## Abstract

Self-injurious behavior (SIB) among adolescents is an important public health issue worldwide. It is still uncertain whether homogeneous subgroups of SIB can be identified and whether constellations of SIBs can co-occur due to the high heterogeneity of these behaviors. In this study, a cross-sectional study was conducted on a large school-based sample and latent class analysis was performed (*n* = 10,069, mean age = 15 years) to identify SIB classes based on 11 indicators falling under direct SIB (DSIB), indirect SIB (ISIB), and suicide attempts (SAs). Social and psychological characteristics of each subgroup were examined after controlling for age and gender. Results showed that a four-class model best fit the data and each class had a distinct pattern of co-occurrence of SIBs and external measures. Class 4 (the baseline/normative group, 65.3%) had a low probability of SIB. Class 3 (severe SIB group, 3.9%) had a high probability of SIB and the poorest social and psychological status. Class 1 (DSIB+SA group, 14.2%) had similar scores for external variables compared to class 3, and included a majority of girls [odds ratio (OR) = 1.94]. Class 2 (ISIB group, 16.6%) displayed moderate endorsement of ISIB items, and had a majority of boys and older adolescents (OR = 1.51). These findings suggest that SIB is a heterogeneous entity, but it may be best explained by four homogenous subgroups that display quantitative and qualitative differences. Findings in this study will improve our understanding on SIB and may facilitate the prevention and treatment of SIB.

## Introduction

Self-injurious behavior (SIB, synonymous with self-harm behavior) refers to a series of behaviors and intention that are performed deliberately and with the knowledge that they can or will result in some degree of physical or psychological injury to oneself [1–3]. SIB is a common risk behavior, and has become a significant global public health issue [4,5]. Epidemiological studies indicated that the prevalence of SIB was 6.9% to 35.6% in adolescents [6–8]. SIB can lead to severe unanticipated harm or premature mortality. Death from SIB is the second leading cause of injury-related death and disability [3,5].

In the broadest sense, SIB includes all kinds of behaviors that can lead to physical and mental self injury. More specifically, SIB is characterized by direct and intentional self-injury that causes body tissue damage [8,9]; damage to oneself and injury to health, as an unintended by-product of activities such as smoking, binge drinking, etc. [2,3]; and suicide attempts (SA, consisting of suicide ideation, suicide plan and suicide behavior) [2,4]. A growing body of research shows that many individuals engage in SIBs using multiple methods (DSIB and/or ISIB), accompanied by various characteristics. For example, some researchers have examined the link between nonsuicidal self-injury (NSSI) and suicidal behavior [7,9]. Some researchers argued that there is a fine line dividing NSSI and SA and distinguishing between the two remains difficult [10,11]. Other researchers aimed to determine the correlates of NSSI, such as smoking and drinking [7,8]. These studies have revealed that SIB includes a wide range of behaviors and intention, and different types of SIB often co-occur. People engaged in self-injury fall into a heterogeneous group. However, the identification of homogeneous subtypes of SIB would be empirically and realistically significant for researchers and clinicians [9,10,12]. Although many studies focused on the examination of differences and similarities among SIBs, such as comparing NSSI with SA, or NSSI+SA with SA alone, or NSSI+SA with no self-harm (NoSH) [9,13,14], these studies used a variable-centered approach and can only distinguish between two or among three types of SIB. However, SIBs are involved in various types of behaviors. For example, if six binary variables are used to delineate various forms of SIB, 64 (2^6^) different response patterns can be obtained from the data, which will be too complex and there are no acceptable solution to analyze them. An alternative method is needed to identify whether these combined SIBs co-occur in different homogeneous subgroups.

Latent class analysis (LCA) is a person-centered approach, which assumes that heterogeneous individuals from a population can be ‘‘typed” or grouped into smaller relatively homogenous subgroups with similar patterns of some behaviors or trait endorsements [15,16]. LCA can also be used to explain associations between a set of observed categorical variables through assumed unobserved, latent classes. Researchers have used this method to explore co-morbidity or to define subtypes based on combinations of emotional and behavioral problems [17–19]. To our knowledge, only a few studies have employed LCA to identify subgroups of individuals who engage in SIBs. For example, three studies identified four classes of self-injurers based on the method, descriptive features, and functions of self-injury [20–22]. Two studies identified three subgroups in terms of frequency, form, function, and age of onset of self-injury, and frequency of self-injury and risk of suicidal behavior, respectively [18,23]. And one study identified two subgroups based on psychopathy and self-injurious thoughts and behaviour [24]. Although these studies have provided strong support for the heterogeneity of SIB manifestations, subgroups were classified by the characteristics of NSSI only or its correlates, rather than the continuum of SIBs. These studies did not examine the potential co-occurrence of different types of DSIB, ISIB, and SA. In addition, participants in these studies were predominately young adults (college students) or adults. Thus, it is imperative to examine behavioral combinations, to identify whether all forms of self-injury can co-occur, and to characterize their presentation among adolescents [4,11,25,26].

In this study, LCA was used to explore whether distinct homogeneous SIB subtypes could be defined and further analyzed the differences and associations among these subtypes, and characterize the co-occurrence of different forms of SIB among junior and senior high-school students. Differences in personality traits and social and psychological characteristics among the identified classes were then examined.

## Materials and Methods

### Subjects

Data were extracted from the National Science-Technology Support Plan Project “national assessment, early-warning and intervention model research on youth risk behavior”, a study of risk behavior and mental health status in junior and senior high-school students. A large-sample multicenter and multistage cross-sectional survey design was used to recruit junior and senior high-school students from March, 2011 to September, 2011. First, ten cities were selected according to the social and economic development level (high, medium, and low) and the representation of mainland China’s geographic administrative divisions (northeastern, northwestern, northern, southeastern, southwestern, and southern). Then, we chose one to three combined junior and senior public high school(s) with medium-level teaching quality and college enrollment rate in each city. Finally, two classes per grade (7^th^–12^th^) were randomly selected at each school.

A total of 13,151 adolescents from 240 classes at 20 schools were recruited in the following 10 cities: Shanghai, Guangzhou, Hangzhou, Suzhou, Changsha, Chengdu, Shenyang, Yinchuan, Cangzhou, and Langfang. Of 11,975 students who completed the questionnaire, 1906 students provided incomplete responses on 11 indicators for LCA or more than 5 items on a scale were excluded. The total sample thus included 10,069 adolescents [4725 (46.9%) males, 5344 (53.1%) females; mean age, 14.79±1.92 (range, 10–20) years] (Table 1 and S1 Table include demographic information pertaining to age, grade, gender, race, family background, and parental education status for subjects and excluded sample). The response rate was 84.93%. Twelve participants whose age data were lost were excluded from the LCA.

**Table 1:** Demographic Characteristics of Subjects.

	Total	Boys (n = 4725)	Girls (n = 5344)	*χ*^*2*^*/t*	*p*
**Age, y (12**^M^**)**	14.79±1.92	14.80±1.92	14.79±1.93	0.48	0.630
**Ethnicity (23** ^M^**)**				1.86	0.173
** Han nationality**	9508(94.6%)	4475(95.0%)	5033(94.4%)		
** Other ethnic minority**	538(5.4%)	237(5.0%)	301(5.6%)		
**Onlyone child (112** ^M^**)**				82.74	< 0.001
** Yes**	6170(62.0%)	3110(66.7%)	3060(57.8%)		
** No**	3787(38.0%)	1554(33.3%)	2233(42.2%)		
**Family composition (188** ^M^**)**					
** Nuclear families**	9084(91.9%)	4276(92.3%)	4808(91.6%)	2.91	0.405
** Divorced families**	394(4.0%)	181(3.9%)	213(4.1%)		
** Single-parent families**	194(2.0%)	87(1.9%)	107(2.0%)		
** Remarried families**	209(2.1%)	87(1.9%)	122(2.3%)		
**Paternal level of education(1038** ^M^**)**				4.03	0.259
** Some primary school**	703(7.8%)	354(8.3%)	349(7.3%)		
** Completed middle school**	3569(39.5%)	1653(39.0%)	1916(40.0%)		
** Completed high school**	2824(32.4%)	1379(32.5%)	1545(32.2%)		
** Completed higher education**	1835(20.3%)	854(20.1%)	981(20.5%)		
**Maternal level of education(1110** ^M^**)**				1.86	0.602
** Some primary school**	1169(13.0%)	568(13.5%)	601(12.6%)		
** Completed middle school**	3803(42.4%)	1774(42.3%)	2029(42.6%)		
** Completed high school**	2541(28.4%)	1183(28.2%)	1358(28.5%)		
** Completed higher education**	1446(16.1%)	667(15.9%)	779(16.3%)		

^M^ missing value (subjects did not state).

### Study Procedure

The study was conducted in accordance with the Declaration of Helsinki and was approved by the Ethics Committee of the Second Xiangya Hospital of Central South University (No: CSMC-2009S167). Prior to participation, legal guardians and adolescents 18 years old and older provided written consent and youth aged 18 years or younger provided assent. The information that the participants can opt-out at any point without giving reasons during the study was included in the informed consent form. Parents were asked to sign the informed consent form if they allow their child to participate in the survey (or leave the form blank if they did not wish their child participate). Together with the informed consent form, the questionnaires and a document describing the study purpose and content were also sent one week before the screening day to the parents or legal guardians of the subjects. To protect the opt in/ opt out families from being identified, the signed consent form and blank (not signed) consent forms were handed in one by one at the teacher's office, instead of collecting at the same time in the classroom. The subjects were asked to report whether their parents or legal guardians were potentially illiterate. No parents were found to use the illiteracy option to opt out of the study. Approximately 5.4% of students in this study were minorities, but all subjects studied and understand Han Chinese language, which was the primary language used in this study. The informed consent form and the document describing the study purpose and content were also prepared in the native language of specific parents or legal guardians who did not understand Han Chinese language. The validity of informed consent forms signed by parents was confirmed by phone call or email. Trained research team members with graduate or undergraduate degrees in psychology administered the questionnaires and explained the study purpose and the items in questionnaires. Students could ask questions to better understand the survey items. Effective strategies were developed to protect the opt in/ opt out families and students from being identified. To maintain confidentiality, all students in a class received the survey, but the researchers asked the students who or whose parents refused to participate in the study just to leave the questionnaires blank, to protect them from being known by their peers. Students were then allowed 25–35 minutes to complete the self-report questionnaires. All data were centralized at the project responsible unit and the quality of questionnaires is ensured by team leaders who randomly visited study sites and verified the proper implementation of questionnaires.

### Measures

#### Health-Risk Behavior Inventory for Chinese Adolescents

SIB was measured by an 11-item questionnaire (see S2 Table), which used items extracted from the Health-Risk Behavior Inventory for Chinese Adolescents (HBICA) that has been validated with good psychometric properties, including test-retest reliability, internal consistency, construct validity, and external validities [27,28]. Responses were dichotomized as “never” (0 score) or “at least once” (1 score). The nomenclatures of SIBs were defined explicitly and primarily. Self-cutting or self-burning, and self-scratching, self-biting, and/or hitting are the typical methods of DSIB, which are conceptualized as direct and intentional self-injury (self-harm) with body tissue damage, irrespective of motive or the extent of suicidal intent [29]. Behaviors such as smoking [29], binge drinking [7], unprotected sexual behavior [30], dangerous driving [26], drug abuse [26], and overeating [26] were categorized into ISIB. Likewise, suicide ideation, suicide plan, and suicide behavior were classified as SAs [2,31–33].

#### Barratt Impulsiveness Scale, Version 11^th^

Impulsivity was measured using the Chinese version of the Barratt Impulsiveness Scale, version 11^th^ (BIS-11) [34]. This widely used 30-item self-report questionnaire has been shown to be reliable and valid among Chinese adolescents [35]. The participants rate the frequency of common activities on a scale ranging from 1 (rarely/never) to 4 (almost always/always). Item scores are summed to obtain an overall impulsiveness score, with higher scores indicating higher levels of impulsivity. In the current study, the Cronbach’s alpha coefficient for the BIS-11 was 0.79.

#### Rosenberg Self-Esteem Scale

Global self-esteem of subjects was acquired using the Chinese version of the 10-item Rosenberg Self-Esteem Scale (RSES) [36], which assesses participants’ self-related “general outlook on life” by measuring positive and negative feelings about themselves. Each item is rated on a four-point scale ranging from 1 (not at all in conformity with me) to 4 (completely in conformity with me). Higher scores indicate higher levels of self-esteem. The internal consistency of the RSES was 0.86 in the present study.

#### Center for Epidemiological Studies Depression Scale

The Center for Epidemiological Studies Depression Scale (CES-D) [37] is a 20-item measure designed to assess depressive symptoms in the general population. Participants’ scores range from 1 (rarely) to 4 (most of the time) with regards to how often they have experienced each symptom in the past week. Total scores range from 20 to 80, with higher scores indicating higher levels of depressive symptoms. The Chinese version of the CES-D has been shown to have good psychometric properties for adolescents [38]. The internal consistency of the scale (Cronbach’s alpha coefficient) was 0.89 in our sample.

#### Multidimensional Anxiety Scale for Children

Anxiety was assessed using the Multidimensional Anxiety Scale for Children (MASC) [39], a 39-item questionnaire designed to assess anxious symptoms in youth. Participants’ scores range from 0 (never applies to me) to 3 (often applies to me) with regards to how often they have experienced each symptom in the past week. Total scores range from 0 to 117, with higher scores indicating higher levels of anxious symptoms [40]. The Cronbach’s alpha coefficient of the MASC was 0.93.

#### Cognitive Emotion Regulation Questionnaire

The Cognitive Emotion Regulation Questionnaire (CERQ) [41] was designed to measure distinct cognitive emotion regulation strategies that individuals may use to cope with emotionally arousing life events. The CERQ is composed of nine subscales and two second-order subscales (adaptive and maladaptive strategies). Responses to the 36 items are structured using a five-point Likert scale ranging from 1 (almost never) to 5 (almost always). A higher subscale score indicated a greater propensity to employ the cognitive strategy in response to adverse or stressful events. The Chinese version of the CERQ has been shown to have good psychometric properties for adolescents [42]. Cronbach’s alpha coefficients for the total scale and the adaptive and maladaptive strategies subscales were 0.68, 0.76, and 0.67, respectively.

#### Adolescent Self-Rating Life Events Checklist

Life events were assessed using the Adolescent Self-rating Life Events Checklist (ASLEC) [43,44], which evaluates the impact of stressful life events experienced during the past 12 months. This instrument lists 26 negative life events, with a response ranging from 0 (did not occur) or 1 (occurred, but no impact at all) to 5 (occurred, extremely severe impact). Higher scores indicate that participants experienced more or more-severe negative life events. Cronbach’s alpha coefficient for the ASLEC was 0.93 in this sample.

### Statistical Analyses

Descriptive analyses were used to examine demographic characteristics and the prevalence of SIBs. The chi-squared test was used to compare the prevalence of SIBs. These statistical analyses were conducted using Predictive Analytics Software (PASW version 18.0).

Following the descriptive analyses, a series of independently estimated LCAs were used to characterize groups of SIBs in adolescents. Eleven dichotomous items were used to define the latent classes. Due to the sampling methods and the nature of the phenomenon of SIB, the local independent assumption may not fulfill in this study. Multilevel latent class models were statistically modeled within school level when the data includes students nested in schools [45]. A series of models were estimated with the number of class ranging from one to six at level 1 (students) and one to three at level 2 (schools). A full information maximum likelihood (FIML) method was employed to handle missing data.

We used several recommended criteria [16] to facilitate model choice, including the Akaike Information Criterion (AIC), Bayesian Information Criterion (BIC), sample-size adjusted Bayesian Information Criterion (ssaBIC), average posterior probabilities, class size, the Lo-Mendell-Rubin’s adjusted likelihood ratio test (LMR-LRT), and entropy. A better-fitting model has a lower BIC and ssaBIC value [46]. The LMR-LRT provides a *p* value, the significance of which indicates whether the k-1 class model is rejected in favor of the k class model. Entropy is an index used to assess the precision of latent class membership assignment; higher probability values indicate greater precision of classification, with a maximum value of 1. The proportions of individuals in each class were also presented. Currently, there is a lack of consensus on which criterion best identifies the best-fitting number of classes [47]. Final class selection is based on the model interpretability and parameter estimations. Following estimation of the optimal number of classes, a set of analyses was conducted to examine potential correlates of each class. Each individual was assigned to a specific class based on the highest posterior probability value for that individual in the retained latent class model.

We conducted a mean difference test with psychological and social correlates [personality traits (impulsivity and self-esteem), emotional status (depression and anxiety), regulation strategies (adaptive and maladaptive), and life events], using one-way analysis of variance, post hoc least significant difference tests, and chi-squared tests, to investigate the relationships between latent classes and auxiliary variables across classes. Multinomial logistic regression analysis was conducted to detect the effects of age and gender on latent class membership. These analyses were conducted using Mplus 7.0 software [48,49].

## Results

### Prevalence of SIBs

Table 2 shows the prevalence of 11 forms of SIB among males, females, and both genders. The prevalence of SIBs in the past 12 months in adolescents ranged from 4.7% (suicide ideation) to 23.2% (self-biting/-scratching/-hitting). Significant differences between genders were found for all items except overeating. Compared to males, females were significantly associated with higher rates of SAs [odds ratios (ORs) = 1.70, 1.63, and 1.51, respectively] and lower rates of smoking, unprotected sexual behavior, and dangerous driving (ORs = 0.32, 0.32, and 0.44, respectively). Among participants who reported engaging in self-cutting or self-burning, 39.2% smoked and 57.7% had suicide ideation, illustrating the high rates of co-occurrence between behavior types.

**Table 2:** Prevalence (%) of SIBs in the Adolescents Stratified by Gender.

	All	*%*	Male	*%*	Female	*%*	*χ2*	*p*	OR	95% CI
**Self-cutting/-burning**	1899	18.9	798	16.9	1101	20.6	22.6	<0.001	1.28	[1.15, 1.41]
**Self-biting/-scratching/-hitting**	2333	23.2	976	20.7	1357	25.4	31.61	<0.001	1.31	[1.19, 1.44]
**Smoking**	1018	10.1	727	15.4	291	5.4	272.68	<0.001	0.32	[0.28, 0.37]
**Binge drinking**	1948	19.3	1114	23.6	834	15.6	102.10	<0.001	0.60	[0.54, 0.66]
**Unprotected sexual behavior**	555	5.5	402	8.5	153	2.9	153.43	<0.001	0.32	[0.26, 0.38]
**Dangerous driving**	1584	15.7	1011	21.4	573	10.7	215.55	<0.001	0.44	[0.40, 0.49]
**Drug abuse**	560	5.6	303	6.4	257	4.8	12.28	<0.001	0.74	[0.62, 0.88]
**Overeating**	1941	19.3	885	18.7	1056	19.8	1.71	0.19	1.07	[0.97, 1.18]
**Suicide ideation**	2147	21.3	788	16.7	1359	25.4	115.53	<0.001	1.70	[1.54, 1.88]
**Suicide plan**	1112	11.0	405	8.6	707	13.2	55.39	<0.001	1.63	[1.43, 1.85]
**Suicide behavior**	473	4.7	177	3.7	296	5.5	18.01	<0.001	1.51	[1.25, 1.82]

SIB = self-injurious behavior, OR = odds ratio, CI = confidence interval.

### Classes Identified through LCA

LCA, conducted with age and gender as covariates, was applied to the 11-items SIBs in a sample of 10,057 students (excluding 12 students with missing information). Parameters of fit and the proportion of individuals in each class were presented (S3 Table). All the fit indices continued to improve with increasing number of classes, with the exception of the entropy index, which was the lowest at two classes. The AIC, BIC, and ssaBIC continued to decrease from one to six classes, but the relative magnitude of the decrease was smaller after the four-class solution. The highest average latent class probabilities (84.6%, 82.3%, 83.9%, and 91.8%, respectively) for each participant’s membership resulted in a four-class solution.

Building of this four-class solution on Level 1, we specified a multilevel latent class model that utilized the parametric approach to account for the nested structure of the data. We estimated from one to three classes parametric random effects models. The BIC shows a much smaller increase from one class to three (72629.1, 72639.7, and 72647.5, respectively). Moreover, entropy is maximized with two classes (0.80) and decreased to 0.44 with three classes. The two-class model appears to be the best model on Level 2 (schools). However, the proportion of school classification were 0.971 vs. 0.029 between the two classes at school level (there are 20 schools, but one of class’s proportion (2.9%) was much less than 5%, which suggested that there was no empirical significance at school level in our study. So we retained the original solution at single level LCA.

**Fig 1:**
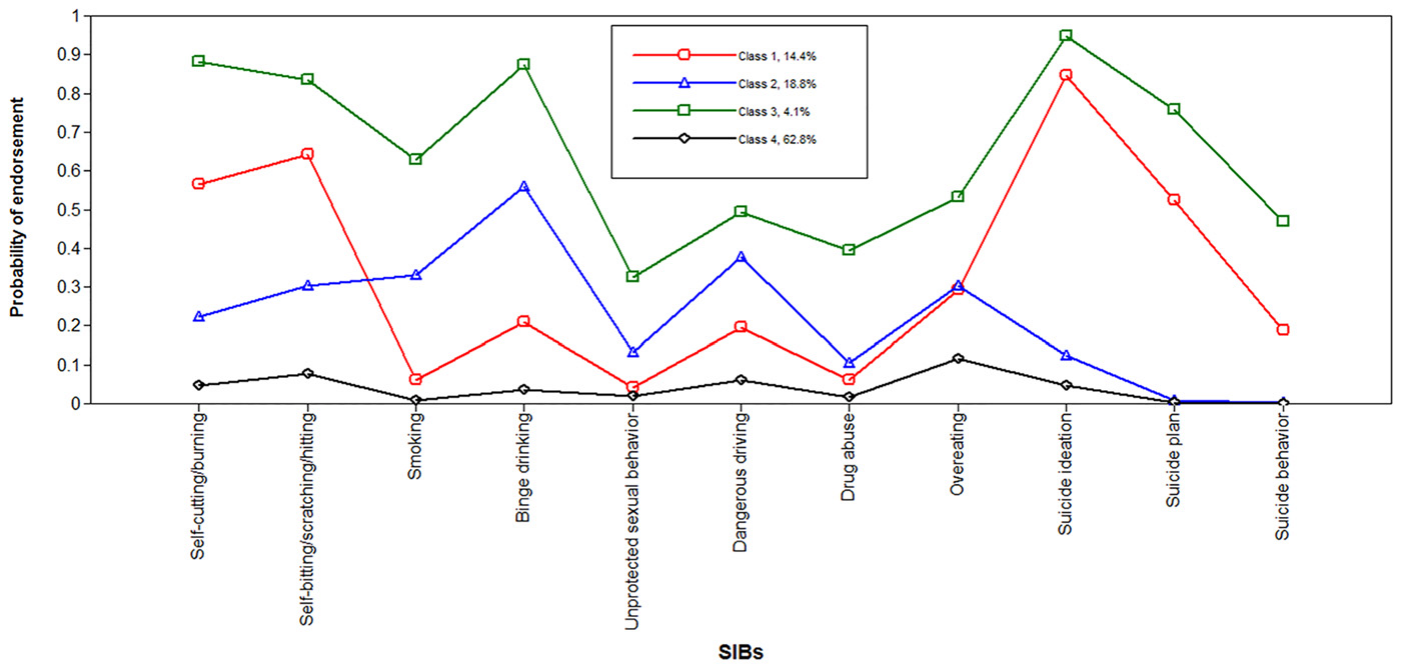
Profiles of 4 SIB classes. Proportions for the latent classes were based on estimated posterior probabilities. Class 1: DSIB+SA group (high endorsement of DSIB and SI, moderate SP; 14.4%); Class 2: ISIB group (low endorsement of DSIB and SI, high ISIB, no endorsement of SP or SB; 18.8%); Class 3: severe SIB group (highest endorsement of all SIBs; 4.1%); Class 4: normative group (lowest endorsement of DSIB and ISIB, no endorsement of SP or SB; 62.8%); SIB: self-injurious behavior; DSIB: direct self-injurious behavior; SA: suicide attempt; SI: suicide ideation; SP: suicide plan; ISIB: indirect self-injurious behavior; SB: suicide behavior; Red: Class 1; Blue: Class 2; Green: Class 3; Black: Class 4.

To our knowledge, a coherent criterion for the identification of the best-fitting model is lacking. According to empirical (goodness of fit statistics, lower BIC and SSABIC with trivial reduction after adding 5 or more classes, and higher entropy), and theoretical (practical interpretability) aspects and the parsimony principle, a four-class model was considered to fit the data best [46,50]. Fig 1 illustrates these latent classes as four distinct groups according to item probabilities.

The first class, named the DSIB+SA group (*n* = 1426, 14.2% prevalence), was characterized by high probabilities of DSIB (> 0.80) and suicide ideation (0.85), a moderate probability (0.52) of suicide plan, and a low probability (< 0.30) of ISIB. The second class, named the ISIB group (*n* = 1666, 16.6% prevalence), showed moderate endorsement of ISIB (probabilities of four ISIB items > 0.30), low endorsement of DSIB (probability ≤ 0.30), and very low endorsement of SA (probability of suicide ideation = 0.12, probabilities of suicide plan and suicide behavior < 0.01). The third class, named the severe SIB group (*n* = 393, 3.9% prevalence), showed the highest endorsement of all items, indicating a severe high-risk profile. The fourth class, named the baseline/normative group (*n* = 6752, 65.3% prevalence), showed the lowest endorsement of all items (probabilities < 0.08 except overeat), indicating a low-risk profile. It’s important to note that the class counts and proportions of each class represent the classification of individuals based on the most likely latent class membership, which was different from proportions based on estimated posterior probabilities (S3 Table).

### Class Differences in Correlates

Table 3 presents the results of analyses of correlates in classes. Significant differences among classes were observed for all correlates. Post hoc comparisons indicated that class 3 had the highest levels of impulsivity, depression, anxiety, and maladaptive strategies; highest frequency of negative life events; and lowest levels of self-esteem and adaptive strategies, whereas class 4 had the complete opposite performance to class 3. Class 1 was fall between class 3 and class 2. Similarities were also observed between classes. Specifically, no significant difference in non-planned impulsiveness, harm avoidance, social anxiety, separation anxiety, cognitive emotional regulation strategies (positive refocusing, self-blame, and rumination), adaptive strategies, or learning-related stress was observed between class 1 and 3. Similarly, no significant difference in separation anxiety, self-blame, and adaptive strategies was found between class 2 and 4. No significant difference in stress related to punishment or loss was observed between class 1 and 2.

**Table 3:** Differences in Correlates among the Four Classes.

	Total Mean **(n = 10069)**	Class 4 Normative group (*n* = 6572)	Class 2 ISIB (*n* = 1666)	Class 1 DSIB+SA (*n* = 1426)	Class 3 Severe SIB (*n* = 393)	a	b	c	d	e	f	*χ*^*2*^
**Age [OR (95% CI)]**		1.00	1.51 (1.44–1.58)	1.11 (1.07–1.15)	1.28 (1.19–1.38)							
**gender [OR (95% CI)]**		1.00	0.23 (0.18–0.29)	1.94 (1.65–2.29)	0.69 (0.48–1.00)							
**Impulsivity**	62.75	60.50	65.35	66.90	70.37	***	***	***	***	***	***	480.06***
**Attention impulsiveness**	16.12	15.83	16.97	17.49	18.70	***	***	***	***	***	***	408.87***
**Motor impulsiveness**	20.29	19.40	21.39	21.74	23.80	0.049	***	***	***	***	***	368.07***
**Non-planned impulsiveness**	26.35	25.72	26.98	27.68	28.23	0.001	0.086	***	***	***	***	113.10***
**Depression**	37.00	34.35	38.54	43.88	46.39	***	***	***	***	***	***	831.56***
**Anxiety**	37.98	34.34	39.32	48.31	51.70	***	0.004	***	***	***	***	467.37***
**Physical**	7.87	6.22	8.65	12.17	14.87	***	***	***	***	***	***	640.75***
**Harm avoidance**	12.91	12.52	13.02	14.14	14.14	***	0.982	***	***	0.001	***	80.94***
**Social anxiety**	10.54	9.40	11.23	13.63	13.89	***	0.459	***	***	***	***	335.98***
**Separation**	6.69	6.22	6.41	8.47	9.01	***	0.098	***	***	0.208	***	209.01***
**Cognitive emotional regulation strategies**												
**Acceptance**	10.89	10.93	11.01	10.58	10.73	0.002	0.500	0.001	0.169	0.468	0.290	12.36**
**Positive refocusing**	11.84	12.10	11.73	11.09	11.17	***	0.726	***	0.009	0.001	***	42.47***
**Refocus on planning**	10.39	10.33	10.64	10.21	10.74	0.004	0.027	0.350	0.648	0.008	0.048	17.08**
**Positive reappraisal**	10.44	10.31	10.61	10.67	10.98	0.711	0.189	0.003	0.095	0.006	0.001	12.93**
**Putting into perspective**	14.40	14.86	13.96	13.41	12.87	***	0.006	***	***	***	***	188.96***
**Self-blame**	11.04	10.79	11.28	11.64	11.61	0.002	0.861	***	***	0.059	***	46.38***
**Catastrophizing**	8.04	7.39	8.55	9.55	10.36	***	0.001	***	***	***	***	310.11***
**Blaming others**	8.90	8.43	9.44	9.84	10.29	0.004	0.037	***	***	***	***	148.95***
**Rumination**	11.43	10.91	11.83	12.74	12.97	***	0.343	***	***	***	***	155.72***
**Adaptive strategy**	57.93	58.52	57.59	55.96	56.49	***	0.496	***	0.047	0.150	0.003	24.95***
**Maladaptive strategy**	39.40	37.52	41.10	43.76	45.20	***	0.021	***	***	***	***	317.19***
**Self-esteem**	30.76	31.68	30.28	28.31	27.47	***	0.013	***	***	***	***	327.38***
**Life events**	31.06	26.67	35.97	39.12	47.68	***	***	***	***	***	***	515.84
**Punishment stress**	5.80	4.81	7.06	7.21	10.15	0.563	***	***	***	***	***	259.22***
**Loss stress**	5.21	4.39	6.15	6.58	8.63	0.062	***	***	***	***	***	210.85***
**Relationship stress**	6.33	5.67	6.89	7.86	8.57	***	0.004	***	***	***	***	263.78***
**Learning stress**	7.94	7.11	8.73	9.87	10.32	***	0.092	***	***	***	***	291.58***
**Adaption stress**	5.83	4.73	7.18	7.68	10.18	0.004	***	***	***	***	***	637.39***

DSIB = direct self-injurious behavior, SA = suicide attempt, ISIB = indirect self-injurious behavior, a = class 1 *vs*. class 2, b = class 1 *vs*. class 3, c = class 1 *vs*. class 4, d = class 2 *vs*. class 3, e = class 2 *vs*. class 4, f = class 3 *vs*. class 4.

*p< 0.05;

**p< 0.01;

***p< 0.001.

CI = confidence interval.

### Class Differences in Covariates (Age and Gender)

Multinomial logistic regression analysis revealed significant differences in age and gender between class 4 (low-risk group) and the other classes (Table 3). Older subjects were more likely to engage in SIB (ORs for classes 1–3 = 1.11, 1.51, and 1.28, respectively) than younger subjects. More girls were in class 1 (OR = 1.94) and more boys were in class 2 and 3 (ORs = 0.23 and 0.69, respectively) compared to class 4.

## Discussion

This was a large, cross-sectional, representative, school-based study on self-injurious behavior in adolescents. Four distinct latent classes were identified, and variation among classes in social and psychological correlates across age and gender were characterized. To our knowledge, this is the only report on the co-occurrence of SIBs and their correlates in adolescents identified by LCA.

This study demonstrated that the prevalence of SIBs in adolescents were various between different type of behaviors, but it is similar to previous reports [6,29,51]. The prevalence of suicide ideation, suicide plan, and suicide behavior is also similar to the reported prevalence in previous studies [52–54]. Consistent with previous reports [53,55], differences in self-harm behavior among adolescents of different genders were observed. The prevalence of DSIB and SA were significantly higher among girls than among boys. The prevalence of ISIBs in the present study is also similar to that found in a population-based study [56], but lower than that in an inpatient study [57]. The gender difference in the prevalence of ISIBs was also reported in these studies. However, there is a lack of consensus on gender differences in SIBs.

In this study, LCA was adopted to identify the appropriate number of distinct latent classes of SIBs based on a large sample size, eleven indicators, and a larger covariates, and identified four quantitatively and qualitatively distinct latent classes. The baseline normative (low-risk) class (class 4) comprised almost two-thirds (65.3%) of the adolescents. Adolescents in class 4 were characterized by the lowest endorsement of all SIB items, with the best mental-health status than adolescents in other three classes. In contrast, adolescents in the smallest group (class 3, the severe SIB group) displayed high levels of endorsement of all SIB items, with the poorest mental-health states. These findings suggested that adolescents with high stress levels and low self-esteem are more likely to engage in SIBs than adolescents with low stress levels and high self-esteem. Likewise, adolescents who engaged in SIBs showed the inability to regulate emotion and tolerate distress. This is not surprising because SIBs have been associated with a variety of clinical disorders characterized by depression, anxiety, low self-esteem, impulsivity, maladaptive emotional regulation strategies, and frequent negative life events [11,33,58–67]. Therefore, measures of impulsivity, depression, anxiety, emotion regulation strategy, self-esteem, and negative life events may distinguish between groups at high and low risk of SIB.

Most interestingly, two intermediate classes emerged from the analysis. A slightly smaller (14.2%) DSIB+SA group (class 1) included adolescents who displayed higher levels of endorsement of items measuring DSIBs and suicide ideation, low probability of ISIB and suicidal behavior, and a moderate probability of suicide plan. The SIB profile in this group is similar to the description of deliberate self-harm, commonly defined as direct and intentional injury to one’s body tissue, regardless of suicide intent [12]. The strength of the majority of correlates for this group was second only to those for the severe SIB group (Class 3), with no difference between these groups in non-planned impulsiveness, harm avoidance, social anxiety, self-blame, rumination, or learning-related stress. However, correlations with other items in the DSIB + SA (class 1) group were superior to those in the severe SIB group (Class 3). These findings suggest a close association between DSIB + SA (class 1) and severe SIB (class 3).

Another intermediate class (ISIB group, class 2) had the same proportion as class 1 (16.6%), displayed an opposite profile to class 1. These adolescents displayed low to moderate levels of endorsement of items measuring DSIBs, high endorsement of ISIB items, slight probability of suicide ideation, and little likelihood of suicide plan and suicide behavior. The characteristics of this group are similar to those of NSSI, which is defined as “self-harm, without the intention of suicide” and classified as a “condition for further study” [68]. In this group, the overall psychological health was worse than that in the baseline/normative group, but superior to that in the severe SIB and DSIB+SA groups. Adolescents in this group also used the same adaptive strategies as adolescents in the baseline/normative group (class 4), and had the same stress levels related to punishment and loss as adolescents in the DSIB+SA group (class 1). However, St Germain and Hooley study reported no difference between individuals prone to DSIB and those likely to engage in ISIB in impulsivity, self-esteem, negative temperament, or depressive symptoms [26]. In addition, St Germain and Hooley [26] asserted that DSIB-prone individuals were more self-critical than those likely to engage in ISIB. In the present study, the DSIB group used more self-blame and rumination strategies than did the ISIB group, with levels similar to those of the severe SIB group (class 3).

In addition, these results strongly suggest that SIB is not a homogenous entity [1,10,14]. Instead, this study suggests the existence of various subtypes of SIB that adolescents may well fall into. These subtypes may include profiles in which individuals display moderate or high levels of one or more of the SIB components. The terms “(deliberate) self-harm” [12,69], “NSSI” [9], “SIB” [2], “self-mutilation” [2], “SA” (or “attempted suicide”), “suicide ideation” (or “suicidal intention”), and “suicidal behavior” [9] have been matters for debate for decades. Our classification could contribute to clarifying the explicitness of this nomenclature and operational definitions to some degree. In common with other findings [70–73], we observed continuums and overlapping behaviors among all forms of self-injury. These findings may be meaningful for research, education, and clinical practice, and they point to the need to recognize adolescents with this range of behaviors, particularly DSIBs due to their association with a high risk of SA [29,74]. Given that the intent of much self-injury and many SAs is ambivalent or undetermined [29], we argue that every adolescent who engages in DSIB, with or without suicidal intention, must be taken seriously. More research is needed to further examine and clarify the trajectories and common correlates of different forms of SIB.

We acknowledged that there are limitations of this study. Firstly, this study used the cross-sectional design, which precluded the determination of causality. Longitudinal data are needed to further explore the relationships between SIBs and their correlates. Secondly, the study relied on self-reported data from native measures to examine SIBs, although the instruments used were standardized and validated. Future research would benefit from using a more detailed data-collection method such as interview, or comparing the native measures with international commonly used scale. Thirdly, a series of behaviors within the broad definition of SIB categories were recorded in dichotomous variables, which were used to determine the LCA solutions. Dichotomous variables have previously been used to record responses to the suicide item of the Beck Depression Inventory to analyze suicidal ideation in depressed college students [75]. However, the use of a full range of responses in SIB may provide more detailed information that could prove beneficial for future studies. Fourth, given that the classroom identifiers were missing from over half the participants, the effect of the classroom could not be estimated in the second level of the sample. Although school level dependencies were not seen in our sample, they could potentially affect the statistical findings. Further study is needed to examine the effect of level dependencies.

## Conclusion

SIB in adolescents can be divided into four distinct subgroups (DSIB+SA, ISIB, severe SIB, and normative group) using LCA. These groups differed qualitatively and quantitatively in terms of impulsivity, self-esteem, emotional status and regulation strategies, and experiences of negative life events. The clarification and definition of four classes may have theoretical and clinical significance in identifying the high risk and low risk of SIB in adolescents.

## Supporting Information

S1 Table The Comparison of Demographic Characteristics between Excluded Sample and the Study Sample. (DOC)

S2 Table The Indicators of Latent Class Analysis which were Extracted from HBICA. (DOC)

S3 Table Parameters of Fit in the Latent Class Analysis. (DOC)
