# Supplementary material for: Four Distinct Subgroups of Self-Injurious Behavior among Chinese Adolescents: Findings from a Latent Class Analysis
Source: PLoS One. 2016 Jul 8;11(7):e0158609. doi: 10.1371/journal.pone.0158609 (PMC4938421; doi:10.1371/journal.pone.0158609)
Supplement: S1 Table — (DOC) [file pone.0158609.s001.doc]

S1 Table. The Comparison of Demographic Characteristics between Excluded Sample and the Study Sample

|  |  | **Excluded sample (n=1906)** | **Study sample (n=10069)** | ***χ^2^/t*** | ***p*** |
| --- | --- | --- | --- | --- | --- |
| **Age, y (15^M^)** |  | 14.60±1.96 | 14.79±1.92 | 4.03 | <.001 |
| **Gender** |  |  |  |  |  |
|  | **Boys** | 965(50.6%) | 4725(46.9%) | 8.81 | 0.003 |
|  | **Girls** | 941(49.4% | 5344(53.1%) |  |  |
| **Ethnicity (29 ^M^)** |  | 6 ^M^ | 23 ^M^ | 0.12 | 0.725 |
|  | **Han nationality** | 1802(94.8%) | 9508(94.6%) |  |  |
|  | **Other ethnic minority** | 98(5.2%) | 538(5.4%) |  |  |
| **Only one child (135^M^)** |  | 23 ^M^ | 112 ^M^ | 0.01 | 0.971 |
|  | **Yes** | 1166(61.9%) | 617062.0%) |  |  |
|  | **No** | 717(38.1%) | 3787(38.0%) |  |  |
| **Family composition (228^M^)** |  | 40 ^M^ | 188 ^M^ | 6.06 | 0.109 |
|  | **Nuclear family** | 1709(91.6%) | 9084(91.9%) |  |  |
|  | **Divorced family** | 751(4.0%) | 394(4.0%) |  |  |
|  | **Single-parent family** | 28(1.5%) | 194(2.0%) |  |  |
|  | **Remarried family** | 54(2.9%) | 209(2.1%) |  |  |
| **Paternal level of education (1248 ^M^)** |  | 210 ^M^ | 1038 ^M^ | 2.55 | 0.467 |
|  | **Some primary school** | 151(8.9%) | 703(7.8%) |  |  |
|  | **Completed middle school** | 669(39.4%) | 3569(39.5%) |  |  |
|  | **Completed high school** | 539(31.8%) | 2942(32.4%) |  |  |
|  | **Completed higher education** | 337(19.9%) | 1835(20.3%) |  |  |
| **Maternal level of education (1339 ^M^)** |  | 229 ^M^ | 1110 ^M^ | 1.00 | 0.802 |
|  | **Some primary school** | 218(13.0%) | 1169(13.0%) |  |  |
|  | **Completed middle school** | 733(43.7%) | 3803(42.4%) |  |  |
|  | **Completed high school** | 462(27.5%) | 2541(28.4%) |  |  |
|  | **Completed higher education** | 264(15.7%) | 1446(16.1%) |  |  |

^M^ missing value (participants did not state).
