# Supplementary material for: Four Distinct Subgroups of Self-Injurious Behavior among Chinese Adolescents: Findings from a Latent Class Analysis
Source: PLoS One. 2016 Jul 8;11(7):e0158609. doi: 10.1371/journal.pone.0158609 (PMC4938421; doi:10.1371/journal.pone.0158609)
Supplement: S2 Table — (DOC) [file pone.0158609.s002.doc]

S2 Table. The Indicators of Latent Class Analysis which were Extracted from HBICA

| No. | Item | **Content (Over the past year)** |
| --- | --- | --- |
| 1 | **Self-cutting/-burning** | Have you ever intentionally hurt yourself by cutting or burning yourself? |
| 2 | **Self-biting/-scratching/-hitting** | Have you ever intentionally hurt yourself by biting, scratching or hitting? |
| 3 | **Smoking** | Have you ever smoked? |
| 4 | **Binge drinking** | Have you ever binged drinking? |
| 5 | **Unprotected sexual behavior** | Have you ever had sexual intercourse? |
| 6 | **Dangerous driving** | Have you ever dangerously driven ignoring consequences? |
| 7 | **Drug abuse** | Have you ever used illegal drugs? |
| 8 | **Overeating** | Have you ever eaten too much or vomited after overeaten? |
| 9 | **Suicide ideation** | Have you ever seriously thought about killing yourself? |
| 10 | **Suicide plan** | Have you ever planned to kill yourself? |
| 11 | **Suicide behavior** | Have you ever tried to kill yourself? |
