# Supplementary material for: Four Distinct Subgroups of Self-Injurious Behavior among Chinese Adolescents: Findings from a Latent Class Analysis
Source: PLoS One. 2016 Jul 8;11(7):e0158609. doi: 10.1371/journal.pone.0158609 (PMC4938421; doi:10.1371/journal.pone.0158609)
Supplement: S3 Table — (DOC) [file pone.0158609.s003.doc]

S3 Table. Parameters of Fit in the Latent Class Analysis

| **C (*n*)** | **P (*n*)** | **Proportions for class based on estimated posterior probabilities** | | | | | | **E** | **AIC** | **BIC** | **ssaBIC** | **VLMR-LRT**  ***p* value** | **BLRT**  ***p* value** |
| --- | --- | --- | --- | --- | --- | --- | --- | --- | --- | --- | --- | --- | --- |
|  |  | 1 | 2 | 3 | 4 | 5 | 6 |  |  |  |  |  |  |
| 1 | 15 | 1.00 | - |  |  |  |  |  | 141,934.2 | 142,042.4 | 141,994.8 | - | - |
| 2 | 25 | 0.23 | 0.77 | - |  |  |  | 0.84 | 75,307.6 | 75,488.0 | 75,408.6 | <0.0001 | <0.0001 |
| 3 | 39 | 0.66 | 0.18 | 0.16 | - |  |  | 0.78 | 73,116.3 | 73,397.7 | 73,273.8 | <0.0001 | <0.0001 |
| **4*** | **53** | **0.14** | **0.19** | **0.04** | **0.63** | **-** |  | **0.79** | **72,246.6** | **72,629.1** | **72,460.7** | **<0.0001** | **<0.0001** |
| 5 | 67 | 0.09 | 0.04 | 0.13 | 0.17 | 0.57 | - | 0.74 | 71,918.6 | 72,402.1 | 72,189.2 | <0.0001 | <0.0001 |
| 6 | 81 | 0.05 | 0.03 | 0.12 | 0.14 | 0.07 | 0.59 | 0.78 | 71,646.1 | 72,230.6 | 71,973.2 | 0.0054 | <0.0001 |

Proportions for the latent classes were based on estimated posterior probabilities. C=latent class, P= free parameter, E=entropy, AIC= Akaike information criterion, BIC=Bayesian information criterion, ssaBIC= sample size-adjusted Bayesian information criterion, VLMR-LRT = Vuong-Lo-Mendell-Rurin likelihood ratio test, BLRT = parametric bootstrapped likelihood ratio test. ^*^Best fitting model.
